# Supplementary material for: SARS-CoV-2 Omicron variant is attenuated for replication in a polarized human lung epithelial cell model
Source: Commun Biol. 2022 Oct 27;5:1138. doi: 10.1038/s42003-022-04068-3 (PMC9610361; doi:10.1038/s42003-022-04068-3)
Supplement: Supplementary file 2 — Supplementary Information [file 42003_2022_4068_MOESM2_ESM.pdf]

Supplementary Information

SARS-CoV-2 Omicron variant is attenuated for replication in a polarized human lung epithelial cell model

Authors

Christin Mache[1], Jessica Schulze[1], Gudrun Holland[2], Daniel Bourquain[3], Jean-Marc Gensch[1], Djin-Ye Oh[1], Andreas Nitsche[3], Ralf Dürrwald[1], Michael Laue[2] and Thorsten Wolff[1 *]

Affiliations

[1]Influenza and other Respiratory Viruses (Unit 17), Dept. of Infectious Diseases, Robert Koch Institute, Seestr. 10, 13353 Berlin, Germany

[2] Advanced Light and Electron Microscopy (ZBS 4), Centre for Biological Threats and Special Pathogens, Robert Koch Institute, Seestr. 10, 13353 Berlin, Germany

[3]Highly Pathogenic Viruses (ZBS 1), Centre for Biological Threats and Special Pathogens, Robert Koch Institute, Seestr. 10, 13353 Berlin, Germany

* Correspondence: Thorsten Wolff, Unit 17 “Influenza and other Respiratory Viruses", Robert Koch Institute, Seestr. 10, 13353 Berlin, Germany. E-mail: wolfft@rki.de

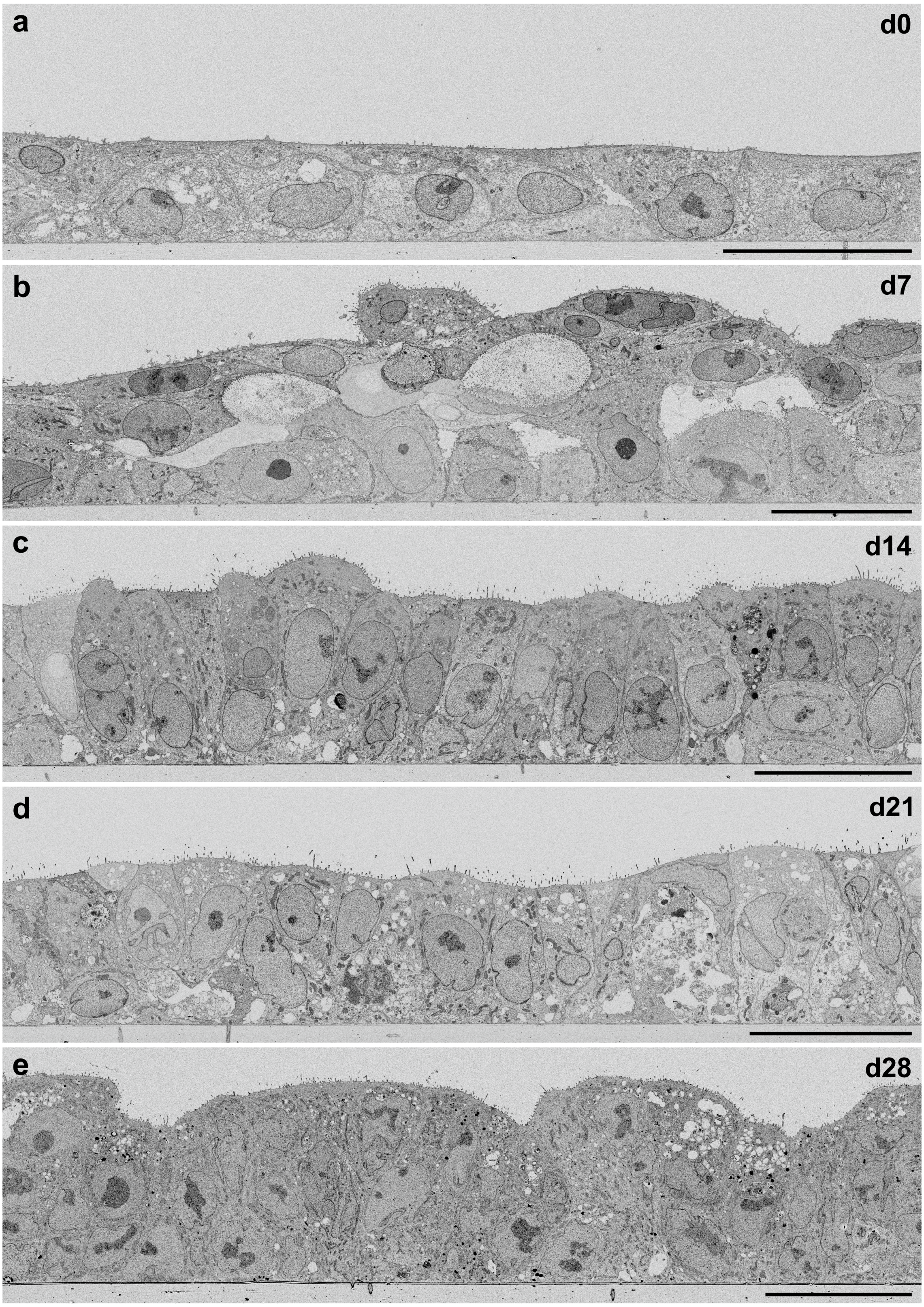

Supplementary Figure 1: EM of cross-sections through non-infected hAELVi-cells at different time points (day 0 to day 28) of cultivation at the ALI. Cells change their shape from cuboidal to columnar and develop into a polarized epithelium, which appears stratified or pseudo-stratified. Images were taken from the block-face by using SEM and detection of backscattered electrons (inverted grayscale). Scale bars = 25 µm.

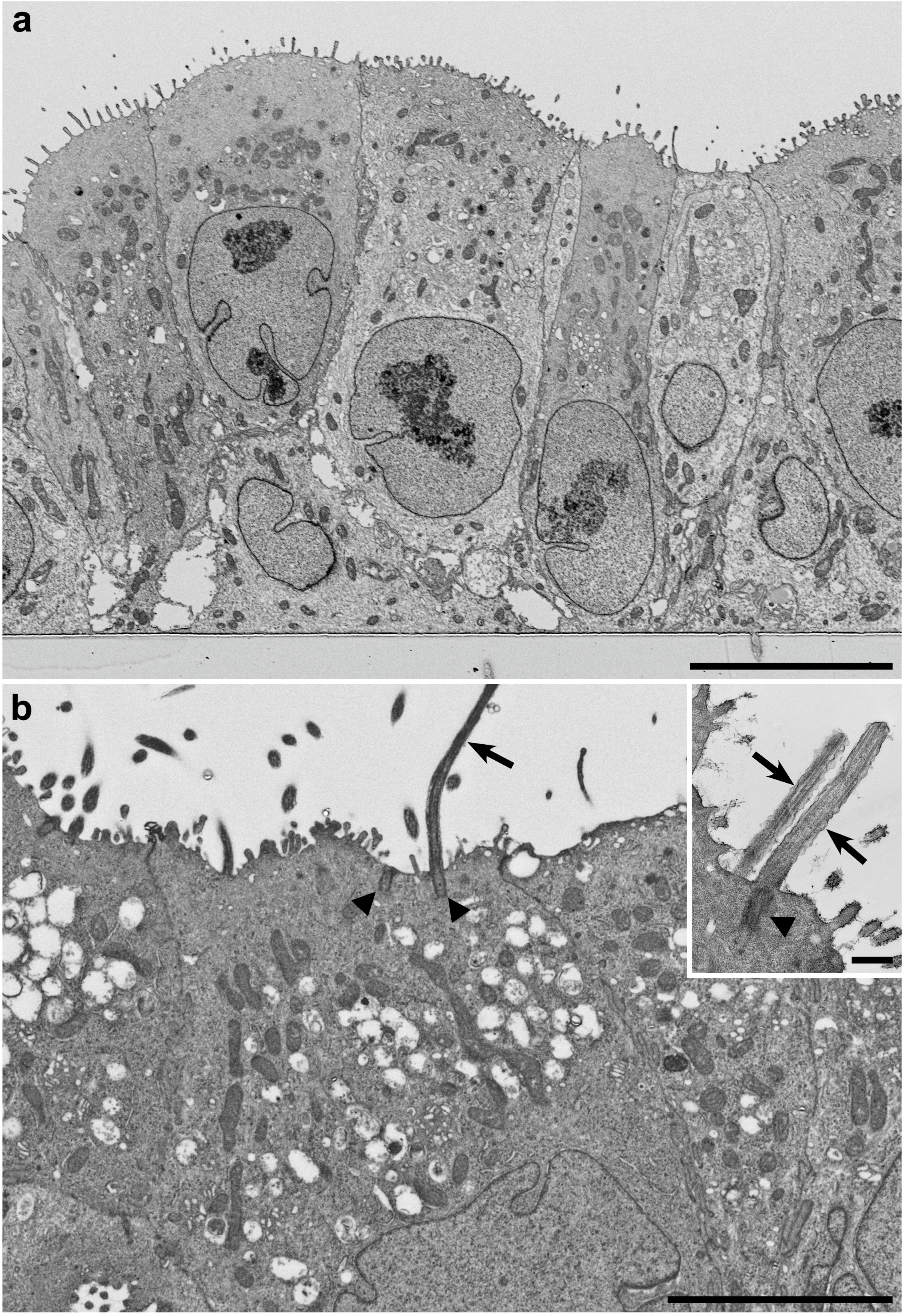

Supplementary Figure 2: EM of the hAELVi-cell epithelium. (a) Cross-section through the hAELVI-cell epithelium on day 14 at ALI. The epithelium appears pseudostratified in certain regions, with cells that have contact with the substrate but do not reach to the apical surface, like basal cells. (b) At later time points, single epithelial cells show cilia (*arrows*) with prominent basal bodies (*arrowheads*) at their surface. Images were taken from the block-face by using SEM and detection of backscattered electrons (inverted grayscale) or (*inset*) from thin sections by TEM. Scale bar in a = 10 µm, b = 5 µm, inset = 0.5 µm.

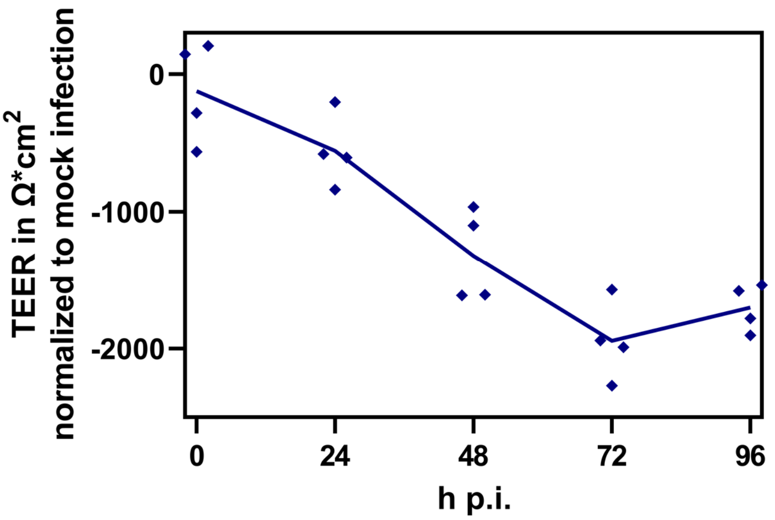

Supplementary Figure 3: Reduction of TEER values upon SARS-CoV-2 infection in hAELVi ALI cultures. hAELVi ALI cultures were infected with SARS-CoV-2 D614G at MOI of 0.3 and TEER values were measured at indicated time points to determine impact of SARS-CoV-2 infection on barrier properties. TEER measurements were performed for n=4 in technical duplicates and data are shown as mean ± SEM normalized to mock-infected samples.

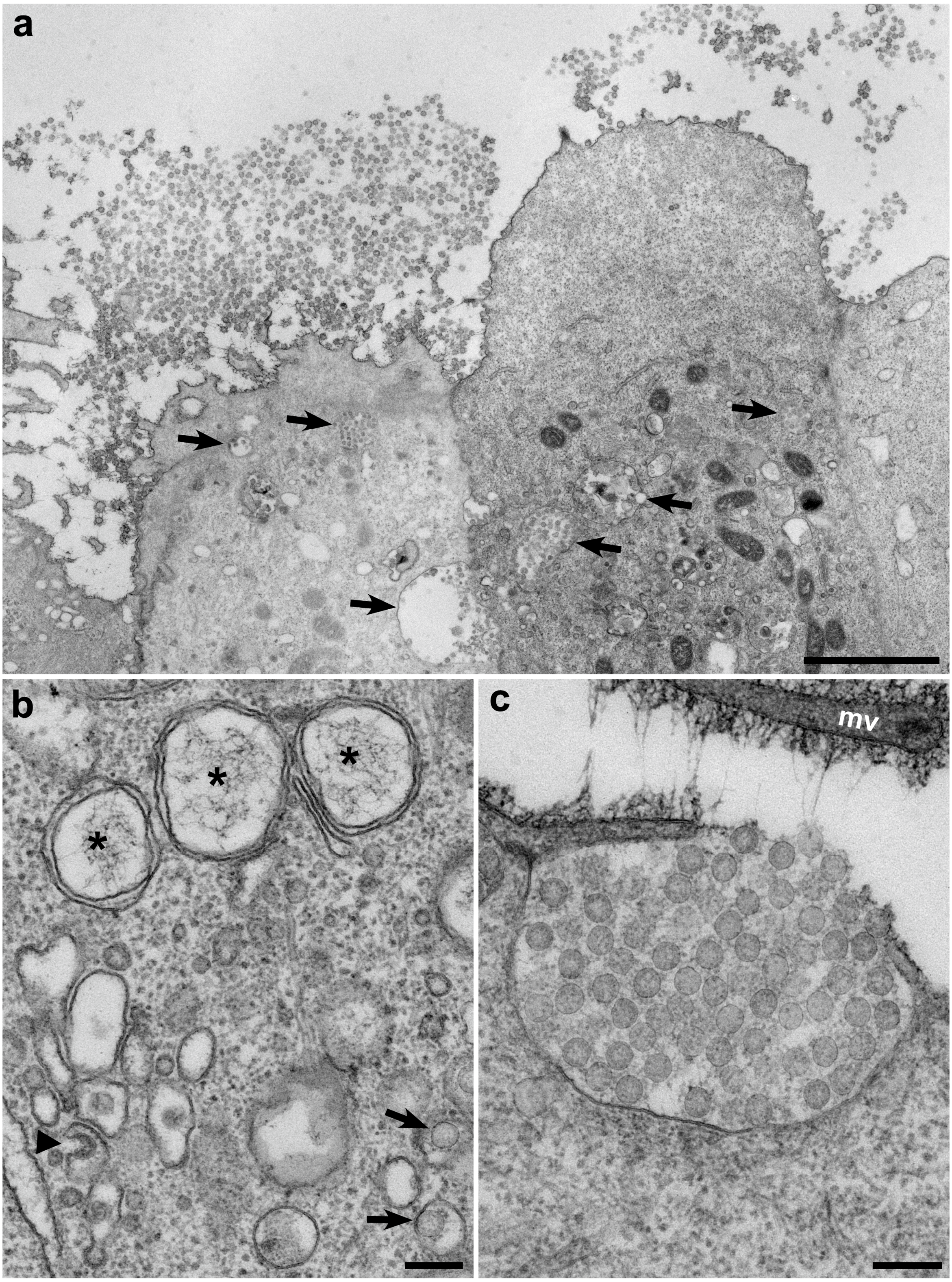

Supplementary Figure 4: EM of hAELVI-cell epithelium infected with SARS-CoV-2 on day 21 at ALI. (a) Apical region of two infected epithelial cells which show large clusters of virus particles at their surface. Larger virus particle assemblies are also visible in membrane-bound compartments within the cells (*arrows*). (b) Double-membrane vesicles with filamentous content (*) or budding structures (*arrowhead*) indicate replication and assembly of virus particles. Single virus particles (*arrows*) are visible in vesicles. (c) Virus particles are released from a large vesicle which has fused with the plasma membrane. The surface of the plasma membrane, including microvilli (*mv*), is coated with filamentous material produced by the cells. Images were taken from thin sections by TEM. Scale bar a = 2 µm, b, c = 200 nm.

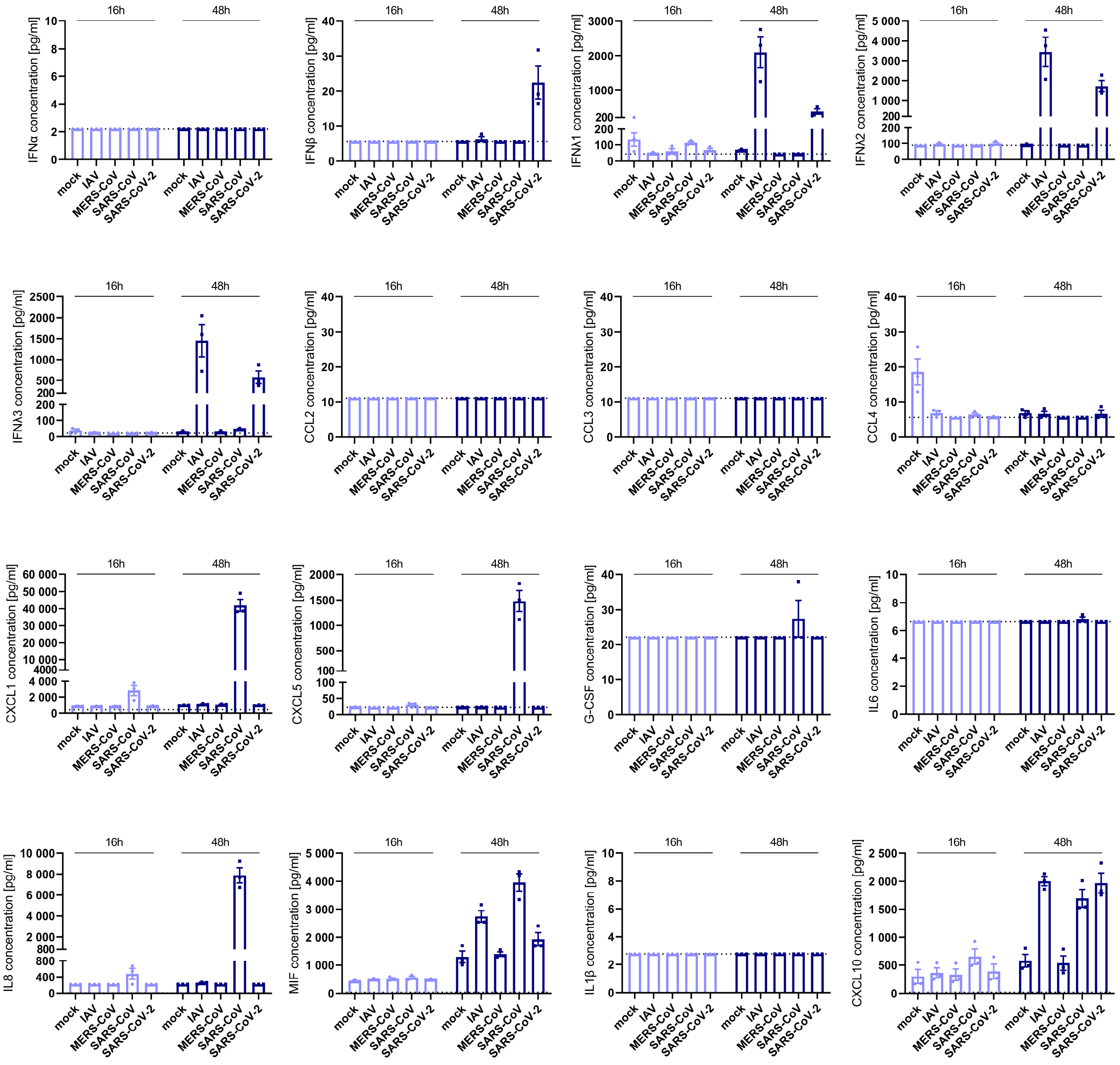

Supplementary Figure 5: **Induction of innate immune factors following virus infection in polarized hAELVi cells.** Cells were infected with SARS-CoV-2, SARS-CoV, MERS-CoV and IAV at MOI of 1 followed by collection of the basolateral fluid at 16h and 48h p.i. for ELISA detection of type I and III IFN or the indicated cyto- and chemokines. Concentrations are shown as mean ± SEM (n=3 in technical duplicates). Limit of detection is marked by the dotted line.

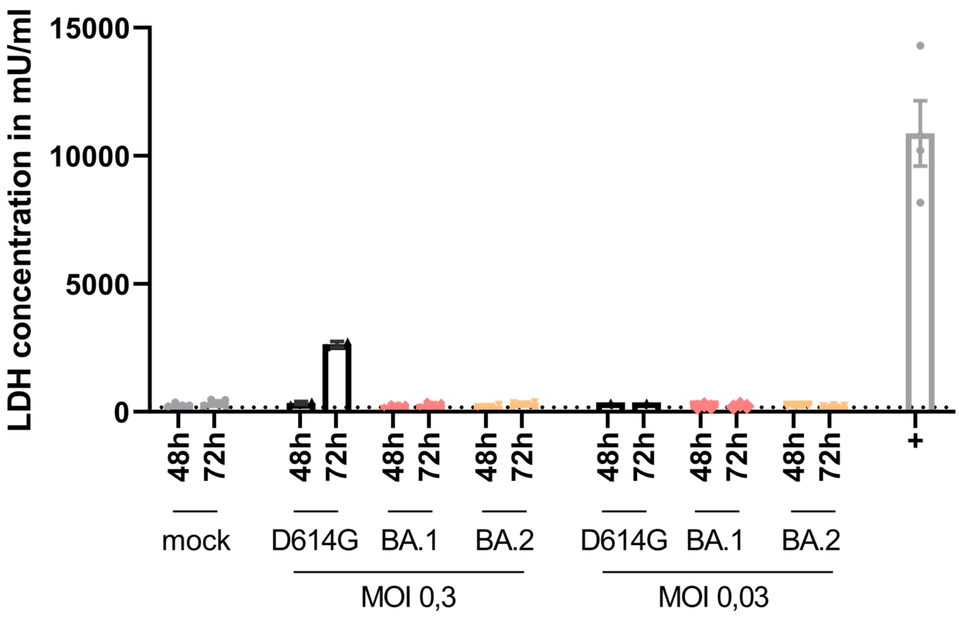

Supplementary Figure 6: Lactate dehydrogenase (LDH) Cytotoxicity Assay of SARS-CoV-2 infected hAELVi ALI cultures. hAELVi cells grown under ALI for 21 days were infected with SARS-CoV-2 variants D641G, Omicron BA.1 or BA.2, respectively, at multiplicities of 0.3 or 0.03 as indicated. To collect samples, apical washes were performed at 37°C at 48h and 72h p.i. for 30 min.. To quantify LDH release, samples were diluted (1:500) and analyzed using a LDH-Glo™ Cytotoxicity Assay (Promega J2381) assay kit according to the manufacturer's instructions. For Maximum LDH Release Control (+), uninfected cells were treated with 2µl of 10% Triton X-100 in DPBS and incubated for 15 min at 37°C. Experiments were performed for n=2 in duplicates.

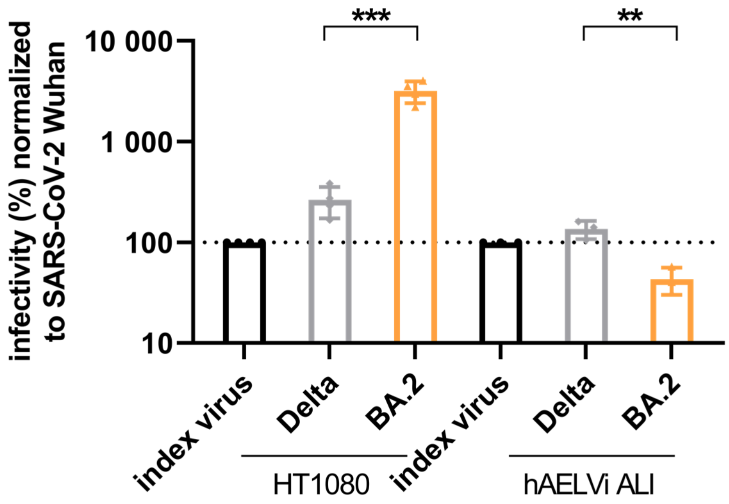

Supplementary Figure 7: Spike-mediated entry into hAELVi ALI cultures using lentiviral pseudo particles. Polarized hAELVi cells were transduced with virus-like particles expressing SARS-CoV-2 spike of Wuhan index virus, Delta or Omicron BA.2, respectively. At 24h p.i. (HT1080) or 48h p.i. (hAELVi) were lysed and NanoLuciferase activity was measured. Infectivity of Delta and Omicron BA.2 was normalized to index virus. Data are shown as mean ± SEM (n=3 in technical duplicates). Dotted line marks 100% (index virus). Statistical analysis was performed using two-sided, unpaired Student's t-test, ** $p<0.01$, *** $p<0.001$.

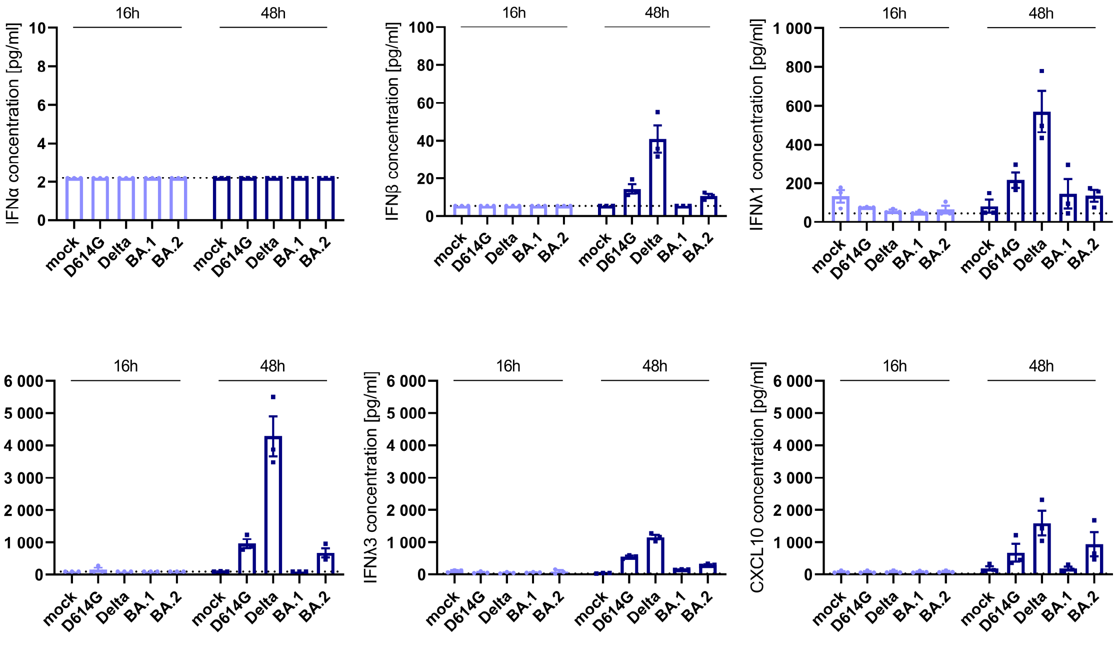

Supplementary Figure 8: Induction of innate immune factors following infection with SARS-CoV-2 VOC in polarized hAELVi cells. Cells were infected with SARS-CoV-2 variants D641G, Delta, Omicron BA.1 or BA.2 at MOI of 1 followed by collection of the basolateral fluid at 16h and 48h p.i. for ELISA detection of type I and III IFN or CXCL10. Concentrations are shown as mean ± SEM (n=3 in technical duplicates). Limit of detection is marked by the dotted line.

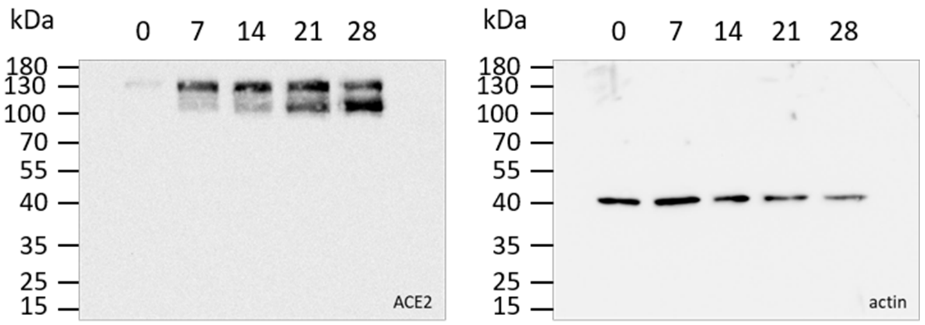

Supplementary Figure 9: Uncropped western blots used in Figure 1D. Western Blot analysis of ACE2 expression of hAELVi cells grown under ALI for 0, 7, 14, 21 or 28 days, respectively.
